# Alteration of Postural Balance in Patients with Fibromyalgia Syndrome—A Systematic Review and Meta-Analysis

**DOI:** 10.3390/diagnostics11010127

**Published:** 2021-01-15

**Authors:** David Núñez-Fuentes, Esteban Obrero-Gaitán, Noelia Zagalaz-Anula, Alfonso Javier Ibáñez-Vera, Alexander Achalandabaso-Ochoa, María del Carmen López-Ruiz, Daniel Rodríguez-Almagro, Rafael Lomas-Vega

**Affiliations:** Faculty of Health Sciences, University of Jaén, Paraje Las Lagunillas s/n, 23071 Jaén, Spain; davidnf15@gmail.com (D.N.-F.); nzagalaz@ujaen.es (N.Z.-A.); ajibanez@ujaen.es (A.J.I.-V.); aaochoa@ujaen.es (A.A.-O.); mlruiz@ujaen.es (M.d.C.L.-R.); dralmagro4@gmail.com (D.R.-A.); rlomas@ujaen.es (R.L.-V.)

**Keywords:** fibromyalgia, chronic fatigue syndrome, postural balance, sensory organization, central sensitization, meta-analysis

## Abstract

Balance problems are one of the most frequent symptoms in patients with Fibromyalgia Syndrome (FMS). However, the extent and nature of this balance disorder are not known. The objective of this work was to determine the best evidence for the alteration of postural balance in patients with FMS and analyze differences with healthy controls. To meet this objective, a systematic review with meta-analysis was performed. A bibliographical search was carried out in PubMed Medline, Scopus, Web of Science, CINAHL and SciELO. Observational studies that assessed postural balance in patients with FMS compared to healthy subjects in baseline conditions, were selected. In a random-effect model, the pooled effect was calculated with the Standardized Mean Difference (SMD) and its 95% confidence interval (CI). Nineteen studies reporting data of 2347 participants (95% female) were included. FMS patients showed poor balance with a large effect on static (SMD = 1.578; 95% CI = 1.164, 1.992), dynamic (SMD = 0.946; 95% CI = 0.598, 1.294), functional balance (SMD = 1.138; 95% CI = 0.689, 1.588) and on balance confidence (SMD = 1.194; 95% CI = 0.914, 1.473). Analysis of the Sensory Organization Test showed large alteration of vestibular (SMD = 1.631; 95% CI = 0.467, 2.795) and visual scores (SMD = 1.317; 95% CI = 0.153, 2.481) compared to healthy controls. Patients with FMS showed worse scores for different measures of postural balance compared to healthy controls. Concretely, FMS patients appear to have poor vestibular and visual scores with a possible somatosensory dependence.

## 1. Introduction

Fibromyalgia syndrome (FMS) is a multi-faceted and chronic disease of unknown etiology [1] characterized by generalized pain tender points and psychosomatic symptoms, such as tiredness, rigidity, sleep perturbations or psychological disorders [2,3]. FMS has been related to increased sensitivity of the Central Nervous System (CNS) to pain marks [4,5]. Widespread pain, headache, muscle spasms and balance disorders are the most prevalent and disabling symptoms [6]. The American College of Rheumatology (ACR) diagnostic criteria, based on the presence of numerous tender points, widespread chronic pain, somatic symptoms and cognitive difficulties, are used to diagnose subjects with FMS [7].

The global mean prevalence of FMS is 2.7% [8] and around 80–90% of diagnosed subjects are middle-aged women [9,10]. Disabling FMS symptoms include physical disabilities that make it difficult to carry out activities of daily living (ADL) and reduce the quality of life [11]. The impact of these symptoms produces an annual cost per diagnosed patient in developed countries of 7256–7900 euros and a related secondary socio-economic burden represented by a high level of absenteeism, unemployment, early retirement and a higher number of sick leave days [12].

Postural stability is a complex task necessary to guarantee the equilibrium and orientation in a gravitational environment [13]. This task requires the rapid and dynamic integration of convergent sensory information from somatosensory, vestibular and visual systems, which are needed to maintain the balance [13,14]. The alteration of the balance can be related to poor balance confidence, increasing the risk of falls [15].

Balance impairments are among the most frequent symptoms in patients with FMS, with a prevalence between 45–68%. Previous studies have shown a correlation between impaired balance and an increased risk of falling and/or a higher frequency of falls in women with FMS with reduced balance compared to healthy subjects [16]. FMS subjects reported an incidence of falls of 1.75 falls per person each six months [15]. Furthermore, balance alterations in patients with FMS negatively impact physical function, gait and quality of life [15,17,18].

There are several studies with different methodologies and very diverse measurement balance tools that make the issue unclear. As we have not detected any review or meta-analysis, the objective of this study was to detect better scientific evidence for the presence of a balance alteration in FMS patients compared to healthy controls. The secondary objective was to analyze which mechanisms of balance would be particularly affected.

## 2. Materials and Methods

### 2.1. Protocol Design

This systematic review with meta-analysis follows the Meta-Analysis of Observational Studies in Epidemiology (MOOSE) Group guidelines [19] and the recommendations contained in the Preferred Reporting Items for Systematic Reviews and Meta-Analyses (PRISMA) statement [20].

### 2.2. Data Sources and Search Strategy

Two authors (D.N.-F. and N.Z.-A.), independently, performed a bibliographic search in PubMed Medline, Scopus, Web of Science, CINAHL and SciELO between September and October 2020. In addition, a search was carried out in the reference lists of retrieved full-text studies and in grey literature. “Fibromyalgia,” “postural balance” and “sensory integration” were the keywords employed in our search strategy in accordance with Medical Subject Headings (MeSH). An expertise author in bibliographical search supported this stage (R.L.-V.). No publication date or language tags were set. Table 1 shows the search strategy used in each database.

### 2.3. Study Selection and Inclusion Criteria

Two blinded reviewers (D.R.-A. and D.N.-F.) independently screened the titles and abstracts of all references to identify the initially selected studies. When one of the authors selected an article in this phase, it was examined in detail. Disagreements that arose during full-text screening were resolved by reference to a third reviewer (R.L.-V.).

The inclusion criteria used were the following: (1) observational studies; (2) studies that evaluated balance in patients with FMS; (3) studies that used balance tests, posturographic or stabilometric measures; (4) studies that included a matched comparison group of healthy controls and (5) studies which assessed balance before any treatment. The exclusion criteria were: (1) observational studies with only one group; (2) studies that did not provide data of the balance assessment to be integrated into the meta-analysis.

### 2.4. Data Extraction

Data of the participants and exposure outcomes from selected studies were collected by two authors (A.J.I.-V. and A.A.-O.) and doubts were resolved by a third author (E.O.-G.). Authorship, publication, research design, total sample size, number of participants in each study and each group (exposed and/or healthy controls), age, sex, body mass index and time since FMS diagnosis were extracted. Balance was the outcome variable. We extracted data (mean and standard deviation) of different tests and posturographic parameters to assess the balance in exposed and healthy controls. We collected data different from the mean and standard deviation susceptible to be transformed and included in the quantitative synthesis [21,22].

### 2.5. Quality Assessment of Studies Included

Two authors (D.N.-F. and M.d.C.L.-R.) independently used the Newcastle-Ottawa Scale (NOS) to evaluate the methodological quality of selected studies [23]. “Selection,” “comparability,” and “ascertainment of exposure” are domains explored by this scale, giving a score from 0 (lowest) to 9 stars (highest) [24]. Studies can be considered with low (0–3 stars), medium (4–6 stars) and high quality (7–9 stars) [25]. According to Meader et al. (2014) [26] and the Grading of Recommendations Assessment, Development and Evaluation (GRADE) system [26], inconsistency was evaluated through the heterogeneity of findings in individual studies (see statistical analysis) and imprecision through the number of included studies (large: >10 studies, medium: 5 to 10 studies and small: <5 studies) and with the median sample size of each study (high: >300 subjects, medium: 100 to 300 subjects and low: <100 subjects) [21,26]. The assessment of publication bias risk is detailed in the statistical analysis.

### 2.6. Statistical Analysis

Two authors performed the statistical analysis (E.O.-G and R.L.-V.) using the Comprehensive Meta-Analysis 3.3.070 (Biostat, Englewood, NJ, USA) [27]. As per Cooper et al., [28] the random-effects model proposed by DerSimonian and Laird was used to generalize the study’s findings [29]. The Cohen’s standardized mean difference (SMD) and its 95% confidence interval (95% CI) were selected to calculate the pooled effect [30]. SMD can be interpreted as small (SMD = 0.2), medium (SMD = 0.5) or large (SMD > 0.8) [31]. Our findings were displayed in the forest plots [32]. Heterogeneity was assessed using the *Q*-test and the degree of inconsistency (I^2^) from Higgins et al. (low < 25%, medium 25–50% and large > 50%) and *p*-value (*p* < 0.1 indicate the presence of heterogeneity) [33,34]. The funnel plots [35] and the Egger test *p*-value (*p* < 0.1 indicates a risk of publication bias) [36] were performed to assess the risk of publication bias. We used the Trim-and-Fill method to estimate the adjusted SMD considering previously any possible publication bias [37].

### 2.7. Sensitivity Analysis

The leave-one-out method was used in the sensitivity analysis to assess the contribution of each study to the pooled effect in each meta-analysis [28].

## 3. Results

### 3.1. Study Selection

**Figure 1:**
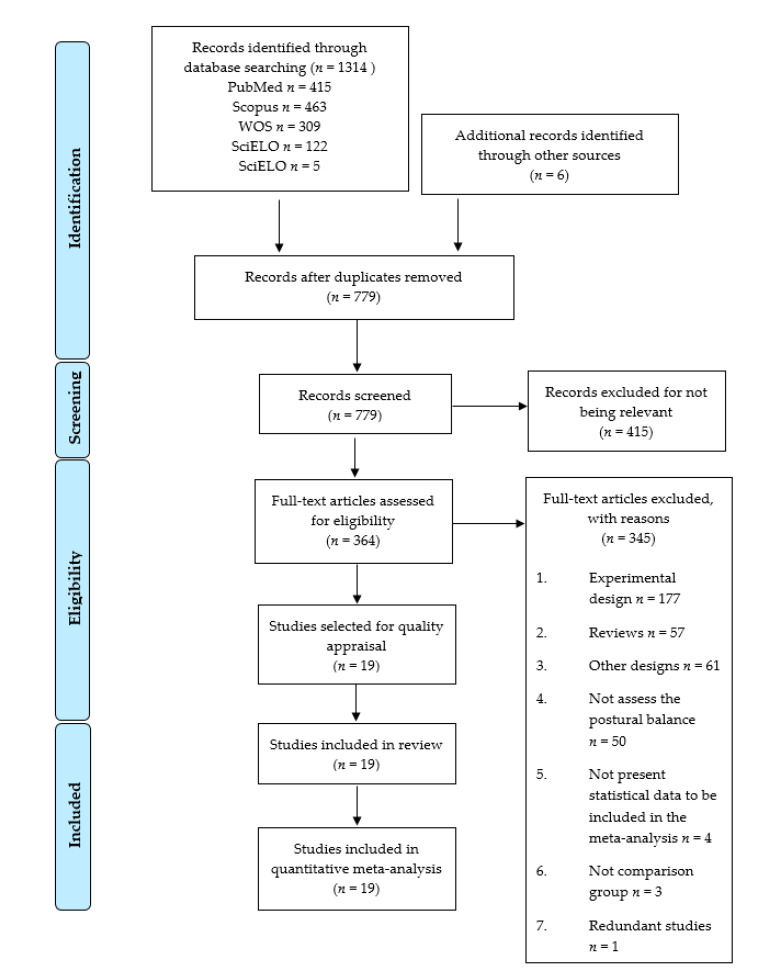
Preferred Reporting Items for Systematic Reviews and Meta-Analysis (PRISMA) flow chart.

The PRISMA flow chart (Figure 1) summarizes the study selection process. The search strategy retrieved 1308 records from the databases and 6 studies were included from the reference lists of the revised articles. Five hundred thirty-five duplicated studies were then deleted and 415 excluded for not being relevant. Three hundred sixty-four full-text studies were assessed for eligibility. Of these, 345 were excluded for not meeting the inclusion criteria (reasons for their exclusion are showed in Figure 1). Finally, 19 studies [38,39,40,41,42,43,44,45,46,47,48,49,50,51,52,53,54,55,56] were included in the review.

### 3.2. Characteristics of the Studies Included in the Review

The main characteristics of the studies included in this review are shown in Table 2. The 19 included studies comprised 49 samples with 49 independent comparisons reporting data from 2347 participants, of which 95% were females (mean age of 44.71 ± 3.77 years old and a mean BMI of 27.03 ± 2.36). The exposed group was composed of 1411 subjects with FMS (46 ± years old and 27.64 ± 2.5 BMI) and 936 healthy subjects (43 ± 4 years old and 26.39 ± 2.09 BMI) formed the control group. All subjects included in this meta-analysis were in the chronic phase. Different balance conditions were assessed: (1) static balance with the One Leg Stance Test; (2) dynamic balance with 8 Foot Up and Go (8FUG) and The Timed Get Up and Go Test (TGUGT); (3) functional balance was assessed with Berg Balance Scale (BBS), MiniBESTest, BESTest and with the balance domain of the Continuous Scale-Physical Functional Performance Test (CS-PFP) and (4) balance confidence with the Activities-Specific Balance Confidence Scale (ABC). Static Posturography was used to detect the Center of Pressure (CoP) sway and dynamic posturography was used to perform the Sensory Organization Test (SOT).

### 3.3. Methodological Quality Assessment

The methodological quality of the included studies assessed by the NOS (Table 3) was medium (NOS mean quality of 4.21 ± 0.78 stars). Three studies [43,44,48] (16% of the total) were scored as low quality and 16 studies [38,39,40,41,42,45,46,47,49,50,51,52,53,54,55,56] (84% of all) were medium quality. A possible risk of selection and confounding bias was present due to the little information provided by the included studies of the characteristics of exposed and non-exposed (S1, S2, S3 and S4 items) and its comparability (C1).

### 3.4. Meta-Analysis of Functional Balance

**Figure 2:**
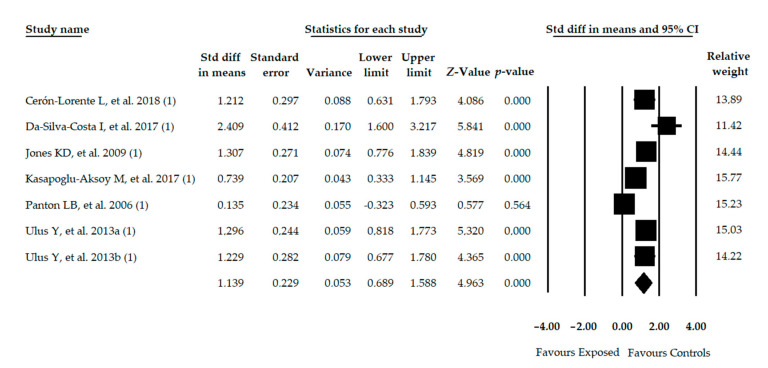
Forest plot of the meta-analysis of functional balance. Std diff, Standardized difference; CI, Confidence interval.

Seven studies [45,46,48,50,51,53,55] were included with 7 independent comparisons reporting data from 493 participants with FMS (46.83 ± 6.29 years old, 98% females and 27.1 ± 1.79 BMI) in which balance was assessed with functional tests. Compared to healthy controls, patients with FMS showing poor functional balance (SMD = 1.138; 95% CI = 0.689, 1.588; *p* < 0.001) (Table 4, Figure 2). Heterogeneity was not high (*I*^2^ = 15%) and the level of precision was low (82.16 participants per study). A funnel plot showed asymmetry and the Egger test (*p* = 0.02) showed a risk of publication bias (Figure S1 in Supplementary Materials). The adjusted pooled effect with Trim-and-Fill method showed a variation of 20% with respect to the original SMD. Sensitivity analysis showed a variation of 14% when compared to the original pooled estimate.

### 3.5. Meta-Analysis of Balance Confidence

**Figure 3:**
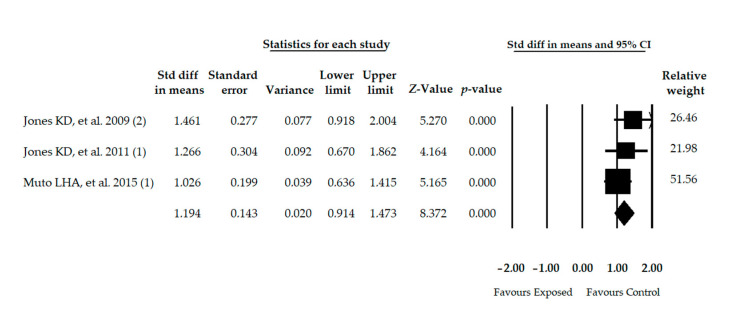
Forest plot of the meta-analysis of balance confidence. Std diff, Standardized difference; CI, Confidence interval.

Three studies [53,54,56] with 3 independent comparisons were included providing data for 235 participants with FMS (47.34 ± 1.83 years old, 96.2% females and 27.69 ± 1.29 BMI) and showing a large effect on balance confidence (SMD = 1.194; 95% CI = 0.914, 1.473; *p* < 0.001) in comparison with healthy controls (Table 4, Figure 3). Heterogeneity was not present and the number of participants per study (78.33) suggested low precision fin-dings. Trim-and-Fill showed a variation of 15% between the adjusted and original effect, suggesting a possible risk of publication bias (Figure S2 in Supplementary Materials). One study removed analysis reported a variation of 14% compared to the original SMD.

### 3.6. Meta-Analysis of Static Balance

**Figure 4:**
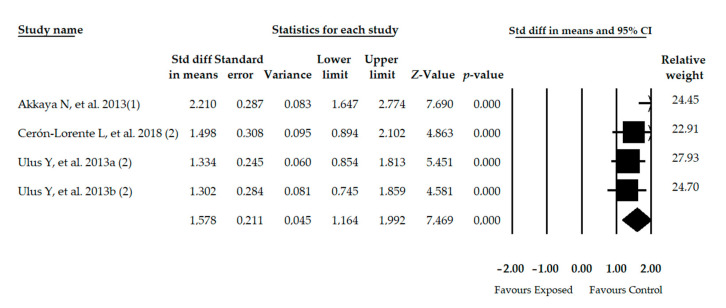
Forest plot of the meta-analysis of static balance. Std diff, Standardized difference; CI, Confidence interval.

Four studies [38,45,46,50] included with 4 independent comparisons reporting data of 286 women with FMS (42.10 ± 6.59 years old and 26.66 ± 0.97 BMI) showed a large effect in the One Leg Stance Test (OLST) (SMD = 1.578; 95% CI = 1.164, 1.992; *p* < 0.001) in comparison with healthy controls (Table 4, Figure 4). Heterogeneity was not present and the precision level was low (71.5 participants per study). The risk of publication bias was not high (Egger *p* = 0.32) and only 6% variation between adjusted and original SMD was found with the Trim-and-Fill method (Figure S3 in Supplementary Materials). Sensitivity analysis provided a variation of 13% compared to the original pooled effect.

### 3.7. Meta-Analysis of Dynamic Balance

**Figure 5:**
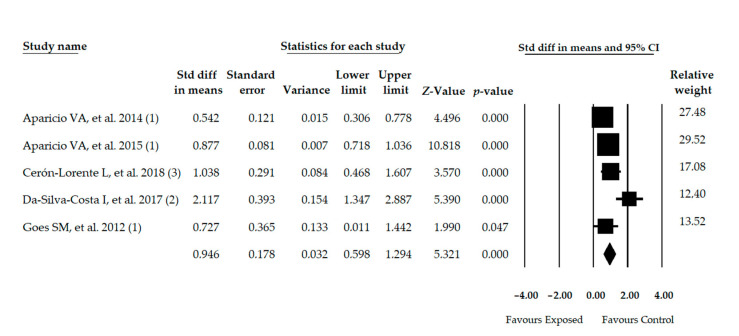
Forest plot of the meta-analysis of dynamic balance. Std diff, Standardized difference; CI, Confidence interval.

Five studies [39,49,50,51,52] were included with 5 independent comparisons providing data of 1,183 women with FMS (48.5 ± 4.92years old and 28.3 ± 3.16 BMI) and showed poor dynamic balance in FMS patients compared to healthy controls (SMD = 0.946; 95% CI = 0.598, 1.294; *p* < 0.001) (Table 4, Figure 5). Moderate heterogeneity was found (*I^2^* = 43.64%) and the precision level of our finding was moderate (236.6 participants per study). The Trim-and-Fill method and Egger test (*p* = 0.23) did not suggest a risk of publication bias (Figure S4 in Supplementary Materials). The sensitivity analysis (leave-one-out method) yielded pooled estimates that varied 18% compared to the original pooled estimate.

### 3.8. Meta-Analysis of Sensory Organization Test with Computerized Dynamic Posturography

**Figure 6:**
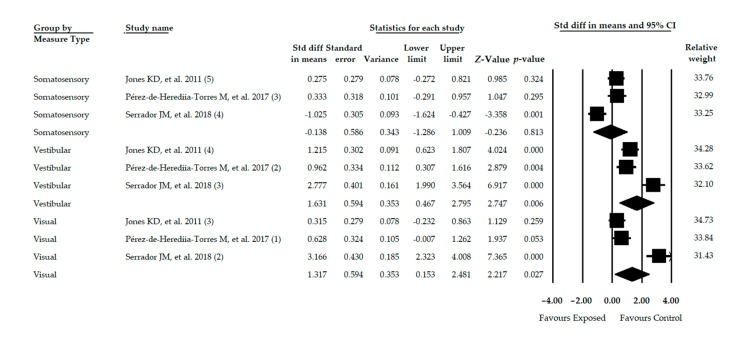
Forest plot of the meta-analysis of Sensory Organization Test. Std diff, Standardized difference; CI, Confidence interval.

Three studies [40,43,53] were included providing data for 423 participants (45.2 ± 0.44 years old, 88% females and 24.71 ± 0.37 BMI). A subgroup analysis was made according to the postural inputs studied. Three independent comparisons assessed the somatosensory score, 3 independent comparisons evaluated the visual score and 3 independent comparisons analyzed the vestibular score. Each subgroup was composed of 141 participants with FMS (80% women with a BMI of 24.69 ± 0.48). In comparison with healthy controls, a large effect on vestibular score was found (SMD = 1.631; 95% CI = 0.467, 2.795; *p* = 0.006) followed by an effect on visual score (SMD = 1.317; 95% CI = 0.153, 2.481; *p* = 0.027) (Table 3, Figure 6). The somatosensory score did not show statistical differences for balance between subjects with FMS and healthy controls (SMD = −0.138; 95% CI = −1.286, 1.009; *p* = 0.813). Heterogeneity was not notable in any subgroups and the level of precision was low (47 participants per study). The risk of publication bias was low in all subgroups and no variations were found with the Trim-and-Fill method.

### 3.9. Meta-Analysis of the Posturographical Parameters of Postural Stability in Bipedal Stance

#### 3.9.1. Eyes Open

**Figure 7:**
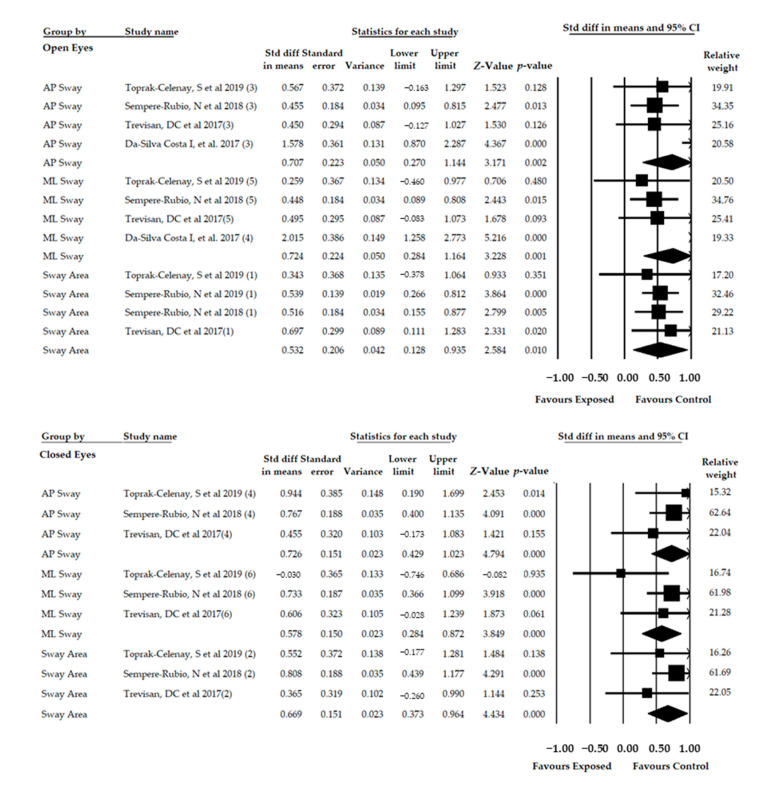
Forest plot of posturographical parameters of Postural Stability in Bipedal Stance. Std diff, Standardized difference; CI, Confidence interval.

Four studies [41,44,47,51] included reporting data for 922 women with FMS (47.70 ± 6.06 years old and 25.82 ± 1.28 BMI). A subgroup analysis was performed according to the posturographic parameter studied; thus, 4 independent comparisons reported the Sway Area parameter (422 women), 4 independent comparisons the AP Sway (250 women) and 4 independent comparisons the ML Sway (250 women). Compared to healthy controls, a moderate effect in Sway Area (SMD = 0.532; 95% CI = 0.128, 2.584; *p* = 0.01), AP Sway (SMD = 0.707; 95% CI = 0.270, 1.144; *p* = 0.002) and ML Sway (SMD = 0.724; 95% CI = 0.284, 1.164; *p* = 0.001) was found in women with FMS (Table 4, Figure 7). Heterogeneity was found in ML Sway subgroup and in this group, the risk of publication bias was important as the adjusted SMD calculated with the Trim-and-Fill method varied 26%.

#### 3.9.2. Eyes Closed

Three studies [41,44,47] were included providing data for 624 women with FMS (47.37 ± 6.22 years old and 25.77 ± 1.31 BMI). Three independent comparisons reported data for the Sway Area parameter (208 women), 4 independent comparisons for the AP Sway (208 women) and 4 independent comparisons for the ML Sway (208 women). A moderate effect was found in all parameters and was higher for AP Sway (SMD = 0.726; 95% CI = 0.429, 1.023; *p* < 0.001), followed by Sway Area (SMD = 0.669; 95% CI = 0.373, 0.964¸ *p* < 0.001) and ML Sway SMD = 0.578; 95% CI = 0.284, 0.872; *p* < 0.001) when compared FMS to healthy subjects (Table 4, Figure 7). Heterogeneity was not present in any subgroups and the level of precision of our results was low (69.33 participants per study). The risk of publication bias was present for Sway Area with a variation of adjusted SMD of 19% with the Trim-and-Fill method.

## 4. Discussion

This systematic review and meta-analysis set out to determine the scientific evidence and examine the existence of a balance alteration in people with FMS compared to healthy controls. Balance has been analyzed using different methods such as static and dynamic posturography, balance confidence and postural tests. Our results showed significant differences between patients with FMS and healthy controls for different balance measurements.

The main symptom of FMS is chronic pain [57], which seems to trigger a process of central sensitization [58]. This is based on an exacerbated response of nociceptive neurons in the CNS to normal (non-painful) affections [59]. Desmeules JA et al. [60] noted that central sensitization was clearly present in FMS subjects. These structural brain changes could affect the correct processing of information from postural afferences and the development of neuromuscular strategies to maintain balance. Central sensitization could be a common factor that explains the relationship found in several studies between lack of balance and some of the most frequent conditions in FMS, such as anxiety, depression or lack of strength [61,62]. These relationships are very frequent in the studies that measured static balance through the various posturographic indices in which the subjects with FMS presented more significant oscillation both with open eyes as with closed eyes, finding associations between worse balance and an increased impact of the disease on factors like fatigue, anxiety, depression, lack of force in inferior members and cognitive symptoms.

Our findings show an alteration in the vestibular and visual scores in comparison to healthy controls. Serrador et al. [43], Pérez-de-Heredia-Torres et al. [40] and Jones et al. [54] reported that the scores for the visual and vestibular scores were significantly lower in the FMS group than in the healthy subjects, with the vestibular scores the most affected. In this sense, Bayazit et al. [63] found an alteration in otolith function in FMS patients without physical alterations of the vestibular system, despite nearly 40% suffering symptoms typical of a vestibular alteration that can be related to distorted balance and worse posture. These same authors observed signs of possible brainstem degeneration, which can be related to poor integration of postural information in the CNS. Therefore, the authors suggest a possible alteration of the vestibular system in FMS patients who have difficulty maintaining balance in the face of a lack of visual and somatosensory information.

Another explanation is offered by the findings of Kuchinand A et al. [64] and Cagnie B et al. [65], who reported a significant decrease in the volume and density of CNS gray matter, specifically in regions related to pain processing (cingulate, insular and prefrontal cortices) and stress (parahippocampal gyrus) in FMS patients. This loss was much greater than that observed in healthy older subjects and therefore suggested that FMS could cause premature CNS aging. All these CNS changes could alter the neural paths of vestibular and visual postural information and be responsible for the distorted balance found in middle-aged FMS patients in the absence of vestibular and visual physical impairments.

Apart from the findings provided by previous studies, patients with FMS may present a balance disorder secondary to drug treatment [66]. Therefore, the search for therapeutic alternatives without adverse effects would be desirable for better management of this problem. Our findings confirm the importance of a balance disorder in patients with FMS, regardless of its origin and how it can be detected with various tests affecting different aspects of postural control. Based on these results, the assessment of patients with FMS should contain a mandatory section for assessing postural balance and the possible alterations of the systems that participate in this complex function.

Additionally, our findings have important implications for clinical practice as balance disorders detected in patients with FMS correlate with the impact of the disease. A review with meta-analysis recently found that the balance disorder presented by patients with FMS can be treated with Training Based on Active Therapy [67]. However, the effect detected in this review is moderate or low and occurs in the short term. Another recent study has confirmed the results of our review, finding that patients with FMS present a specific alteration of balance when the visual and vestibular systems are challenged [68]. These findings support the implementation of treatment programs based on Vestibular Rehabilitation instead of the usual approach based more on proprioceptive exercise.

Some limitations can be found in this review. First, the low number of studies included in each meta-analysis and the low sample size in each study can make the generalization of our findings difficult. Second, the low-medium methodological quality of the studies included, as assessed with NOS, especially in the selection items may induce a possible risk of selection bias. Third, the possible risk of publication bias found in some meta-analyses may underestimate the original pooled effect. In addition, some of the studies found in literature were not included as these did not provide statistical data that were able to be included in this meta-analysis, potentially contributing to the presence of risk of publication bias.

## 5. Conclusions

Patients with FMS showed worse scores for static monopedal balance, dynamic and gait balance, functional balance tests and static posturography parameters compared to healthy controls. In addition, in the Sensory Organization Test, patients with FMS showed worse vestibular and visual scores with possible somatosensory dependence compared to healthy participants. With these data, future studies should explore the efficacy of Vestibular Rehabilitation for patients with FMS.

## Figures and Tables

**Table 1:** Bibliographic search strategy used in each database.

Databases	Search Strategy
PubMed Medline	(fibromyalgia[mh] OR fibromyalgia[tiab] OR fibromyalgias[tiab] OR fatigue syndrome, chronic[mh] OR [tiab]) AND (postural balance[mh] OR postural balance[tiab] OR balance[tiab] OR balance, postural[tiab] OR equilibrium[tiab] OR musculoskeletal equilibrium[tiab] OR postur*[tiab] OR proprioception[mh] OR propriocept*[tiab] OR sensory integration[tiab])
SCOPUS	(TITLE-ABS-KEY (“fibromyalgia” OR “chronic fatigue syndrome”) AND TITLE-ABS-KEY (“balance” OR “equilibrium” OR “postural balance” OR “sensory integration”))
Web of Science	TITLE: (*chronic fatigue syndrome* OR *fibromyalgia*) AND TOPIC: (*balance* OR *postural balance* OR *sensory integration*)
CINAHL	(MH fibromyalgia OR AB fibromyalgia OR AB chronic, fatigue syndrome OR AB fatigue chronic syndrome) AND (MH balance, postural OR AB balance postural OR AB balance OR postural balance OR AB sensory integration)
SciELO	(fibromyalgia OR chronic fatigue syndrome) AND (postural balance OR balance OR equilibrium)

**Table 2:** Characteristics of the studies included in the review.

				Fibromyalgia Group				Healthy Group				Outcome	
Author and Year	Country	K	N	N_e_	Age (Mean ± SD)	% Fem	BMI (Mean ± SD)	N_c_	Age (Mean ± SD)	% Fem	BMI (Mean ± SD)	Balance Condition	Assessment Tools
Akkaya, N et al. (2013) [38]	Turkey	1	80	48	35.9	100%	27.8	32	33.2	100%	25.8	Static	OLST
Aparicio, VA et al. (2014) [39]	Spain	1	316	208	53.9	100%	34.4	108	52.3	100%	33.5	Dynamic	8FGT
Aparicio, VA et al. (2015) [49]	Spain	1	737	487	51.9	100%	28.6	250	49.3	100%	26.5	Dynamic	8FGT
Cerón-Lorente, L et al. (2018) [50]	Spain	3	56	34	52.8	100%	27.6	22	50.1	100%	25.3	-Functional -Static -Dynamic	-MiniBESTest -OLST -TGUGT
Da-Silva-Costa, I et al. (2017) [51]	Portugal	4	42	26	49.2	100%	26.5	16	43.5	100%	25.5	-Functional -Dynamic -Static (Posturography)	-BBS -TGUGT -AP/ML Sway
Goes, SM et al. (2012) [52]	Brazil	1	32	16	41.5	100%	28.1	16	40.4	100%	26.7	Dynamic	TGUGT
Jones, KD et al. (2009) [53]	United States	2	66	34	47.4	88%	30.4	32	46.5	100%	25.5	-Functional -Confidence	-BESTest -ABC
Jones, KD et al. (2011) [54]	United States	4	52	25	50.8	88%	-	27	46.5	93%	-	-Confidence -Dynamic (Posturography)	-ABC -SOT
Kasapoglu-Aksoy, M et al. (2016) [55]	Turkey	1	100	53	46.4	96%	27.5	47	44.4	87%	26.4	Functional	BBS
Muto, LHA et al. (2015) [56]	Brazil	1	117	67	49	100%	27.7	50	43	100%	26.4	Confidence	ABC
Panton, LB et al. (2006) [48]	United States	1	79	29	46	100%	31.1	50	64.5	100%	26.8	Functional	CS-PFP
Pérez-de-Heredia-Torres, M et al. (2017) [40]	Spain	3	40	20	48	100%	24.2	20	47	100%	23.8	Dynamic (Posturography)	SOT
Sempere-Rubio, N et al. (2018) [41]	Spain	6	129	80	53.9	100%	26.9	49	54.4	100%	25.9	Static (Posturography)	Ellipse/AP/ML Sway
Sempere-Rubio, N et al. (2019) [42]	Spain	1	223	123	54.4	100%	-	100	54.2	100%	-	Static (Posturography)	Ellipse
Serrador, JM et al. (2018) [43]	United States	3	49	27	40.3	77%	25.1	22	38.6	77%	25.7	Dynamic (Posturography)	SOT
Toprak-Celenay, S et al. (2019) [47]	Turkey	6	30	15	39.7	100%	24.6	15	39	100%	23.8	Static (Posturography)	Ellipse/AP/ML Sway
Trevisan, DC et al. (2017) [44]	Italy	6	49	29	48.3	100%	26.3	20	48.9	100%	27.4	Static (Posturography)	Ellipse/AP/ML Sway
Ulus, Y et al. (2013a) [45]	Turkey	2	90	60	42.3	100%	27.3	30	40.6	100%	25.9	-Functional -Static	-BBS -OLST
Ulus, Y et al. (2013b) [46]	Turkey	2	60	30	41	100%	26.0	30	40.6	100%	27.3	-Functional -Static	-BBS -OLST

Abbreviatons: K, Number of comparisons; N, Total sample size; N_e_, Exposed sample size; SD, Standard deviation; % Fem, Percentage of women; N_c_, Controls sample size; BMI, Body mass index; OLST, One leg stance test; 8FUG, 8 foot get up test; TGUGT, Timed get up and go test; BBS, Berg balance scale; AP, Antero-posterior; ML, Medio-lateral; ABC, Activities-Specific balance confidence scale; SOT, Sensory organization test; CS-PFP, Continuous scale-physical functional performance test.

**Table 3:** Newcastle-Ottawa Scale (NOS) scores for the methodological quality assessment of included studies.

Study	S1	S2	S3	S4	C1	E1	E2	E3	Total
Akkaya, N et al. (2013) [38]	*	*	-	-	**	-	-	-	4
Aparicio, VA et al. (2014) [39]	*	*	-	-	**	-	-	-	4
Aparicio, VA et al. (2015) [49]	*	*	-	-	**	-	-	-	4
Cerón-Lorente, L et al. (2018) [50]	*	*	-	-	**	-	-	-	4
Da-Silva-Costa, I et al. (2017) [51]	-	*	*	-	**	-	-	-	4
Goes, SM et al. (2012) [52]	*	*	*	-	**	-	-	-	5
Jones, KD et al. (2009) [53]	-	*	*	*	**	-	-	-	5
Jones, KD et al. (2011) [54]	-	*	*	*	**	-	-	-	5
Kasapoglu-Aksoy, M et al. (2017) [55]	*	*	-	*	**	-	-	-	5
Muto, LHA et al. (2015) [56]	-	*	*	-	**	-	-	-	4
Panton, LB et al. (2006) [48]	-	-	*	-	**	-	-	-	3
Pérez-de-Heredia-Torres, M et al. (2017) [40]	*	*	*	*	**	-	-	-	6
Sempere-Rubio, N et al. (2018) [41]	*	*	-	-	**	-	-	-	4
Sempere-Rubio, N et al. (2019) [42]	*	*	-	-	**	-	-	-	4
Serrador, JM et al. (2018) [43]	-	*	-	-	**	-	-	-	3
Toprak-Celenay, S et al. (2019) [47]	*	*	-	*	**	-	-	-	5
Trevisan, DC et al. (2017) [44]	-	*	-	-	**	-	-	-	3
Ulus, Y et al. (2013a) [45]	*	*	-	-	**	-	-	-	4
Ulus, Y et al. (2013b) [46]	*	*	-	-	**	-	-	-	4

Each study can be awarded a maximum of one star for each numbered item within the Selection (S) and Exposure (E) categories. A maximum of two stars can be given for Comparability (C). S1, Adequate case definition; S2, Representativeness of the cases; S3, Selection of controls; S4, Definition of controls; C1, Comparability of cases and controls; E1, Ascertainment of Exposure; E2, Same method of ascertainment for cases and controls; E3, Non-Response rate; “*”, 1 Star or 1 point; “**”, 2 stars or 2 points; “-“ no stars or 0 points.

**Table 4:** Main findings in meta-analyses.

	Pooled Effect						Publication Bias			Heterogeneity		
	K	N	N_s_	SMD	95% CI	*p*-Value	Funnel Plot (Egger *p*-Value)	Trim-and-Fill		Q-test	*I*^2^ (%)	*p*-Value
								Adj SMD	% of var			
Functional balance												
Overall	7	493	82.1	1.14	[0.689, 1.588]	<0.001	*p* = 0.02	0.91	20%	7.07	15.23	0.31
Confidence balance												
Overall	3	235	78.3	1.19	[0.914, 1.473]	<0.001	*p* = 0.18	1.03	15%	1.7	0	0.42
Static balance												
Overall	4	286	71.5	1.58	[1.164, 1.992]	<0.001	*p* = 0.32	1.66	5%	3.01	0.57	0.38
Dynamic balance												
Overall	5	1,183	236.6	0.94	[0.598, 1.294]	<0.001	*p* = 0.23	0.94	0%	7.09	43.65	0.13
Sensory Organization (Dynamic Posturography)												
Vestibular	3	141	47	1.63	[0.467, 2.795]	0.006	*p* = 0.16	1.63	0%	2.34	14.59	0.31
Visual	3	141	47	1.32	[0.153, 2.481]	0.027	*p* = 0.14	1.31	0%	2.56	22	0.27
Somatosensory	3	141	47	−0.14	[−1.286, 1.009]	0.813	*p* = 0.42	−0.14	0%	2.03	1.53	0.36
Postural stability in bipedal stance. Eyes Open (Static Posturography)												
Sway Area	4	422	105.5	0.53	[0.128, 2.584]	0.01	*p* = 0.44	0.55	3%	0.28	0	0.96
AP Sway	4	250	62.5	0.71	[0.270, 1.144]	0.002	*p* = 0.20	0.81	14%	3.99	24.87	0.26
ML Sway	4	250	62.5	0.72	[0.284, 1.164]	0.001	*p* = 0.24	0.92	26%	8.07	62.84	0.04
Postural stability in bipedal stance. Eyes Closed (Static Posturography)												
Sway Area	3	208	69.3	0.67	[0.373, 0.964]	<0.001	*p* = 0.39	0.8	19%	1.97	0	0.57
AP Sway	3	208	69.3	0.73	[0.429, 1.023]	<0.001	*p* = 0.46	0.72	0%	1.08	0	0.58
ML Sway	3	208	69.3	0.58	[0.284, 0.872]	<0.001	*p* = 0.38	0.58	0%	2.08	4.26	0.35

Abbreviations: K, Number of studies; N, Number of participants in each meta-analysis; N_s_, Mean number of participants per study; SMD, Standardized Mean Difference; CI, Confidence Interval; *I^2^*, Higgins Degree of inconsistency; Adj, Adjusted; % of var, Percentage of variation.

## Supplementary Materials

The following are available online at https://www.mdpi.com/2075-4418/11/1/127/s1, Figure S1: Funnel plot of the meta-analysis of functional balance; Figure S2: Funnel plot of the meta-analysis of confidence balance; Figure S3: Funnel plot of the meta-analysis of static balance and Figure S4: Funnel plot of the meta-analysis of dynamic balance.
