# Supplementary material for: Alteration of Postural Balance in Patients with Fibromyalgia Syndrome—A Systematic Review and Meta-Analysis
Source: Diagnostics (Basel). 2021 Jan 15;11(1):127. doi: 10.3390/diagnostics11010127 (PMC7830486; doi:10.3390/diagnostics11010127)
Supplement: Supplementary file 1 [file diagnostics-11-00127-s001.pdf]

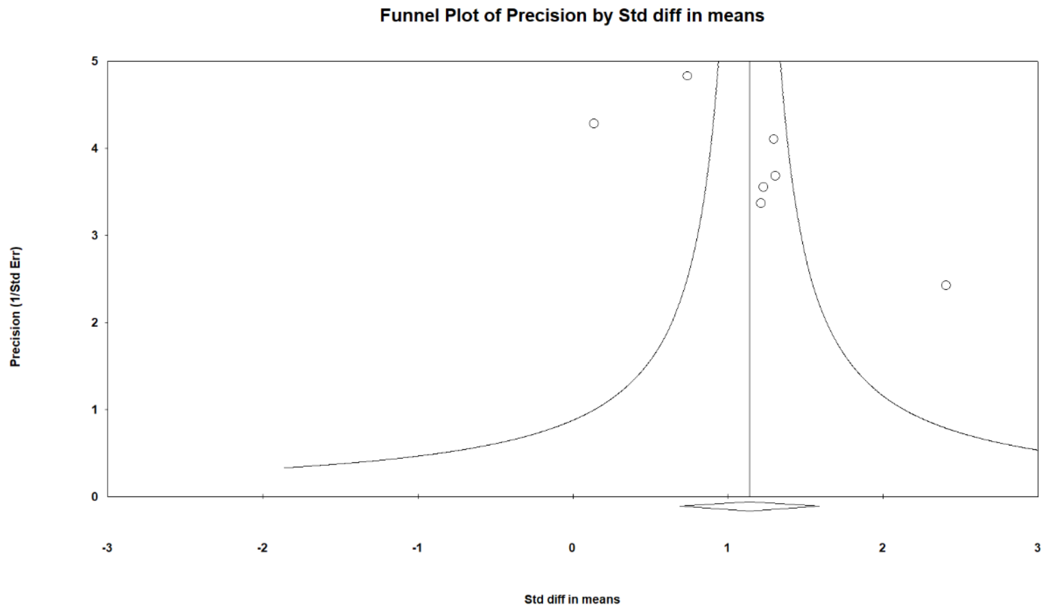

Figure S1. Funnel plot of the meta-analysis of functional balance

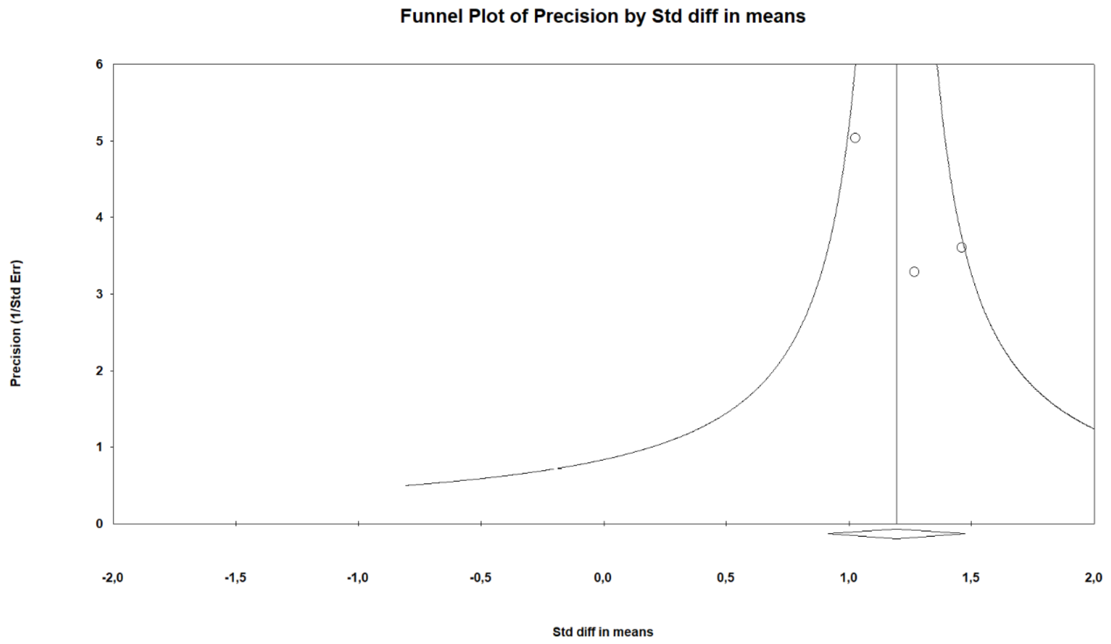

Figure S2. Funnel plot of the meta-analysis of confidence balance

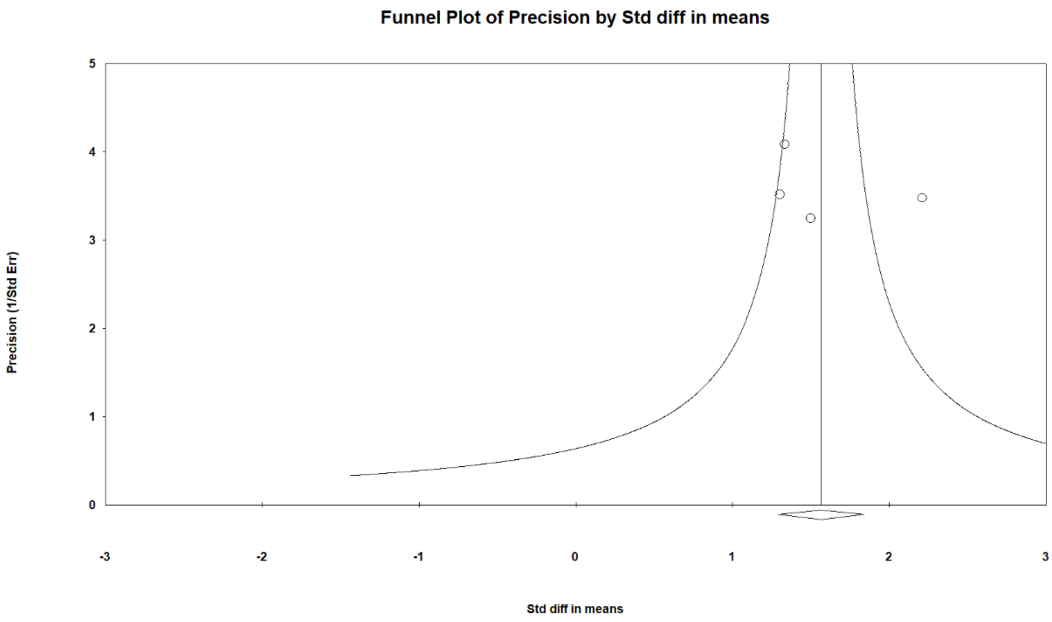

Figure S3. Funnel plot of the meta-analysis of static balance

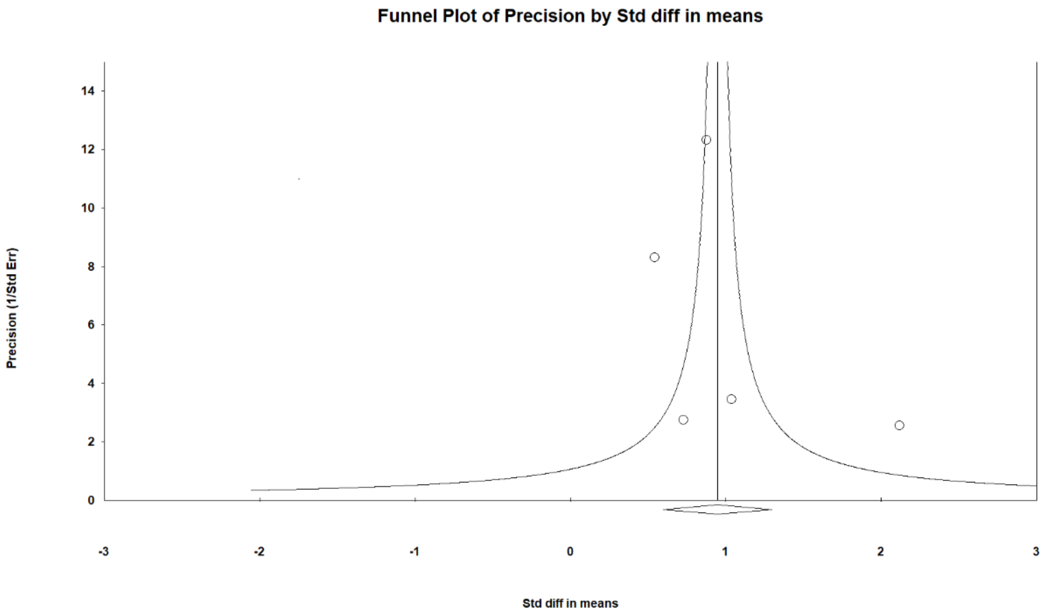

Figure S4. Funnel plot of the meta-analysis of dynamic balance
